# Isolated lumbar intradural tailgut cyst: A case report and review of the literature

**DOI:** 10.1016/j.heliyon.2021.e08223

**Published:** 2021-10-21

**Authors:** Antonio Colamaria, Matteo Sacco, Giovanni Parbonetti, Maria Blagia, Francesco Carbone, Matteo de Notaris

**Affiliations:** a Division of Neurosurgery, “Policlinico Riuniti” Hospital, 1 Viale Luigi Pinto, 71122 Foggia, Italy; b Division of Neurosurgery, “San Pio” Hospital, 53 Via Pacevecchia, 82100 Benevento, Italy; c Division of Neurosurgery, “Giovanni XXIII” Hospital, 207 Via Giovanni Amendola, 70126 Bari, Italy; d Faculty of Medicine and Surgery, University of Foggia, 121 Via Napoli, 71122 Foggia, Italy

**Keywords:** Tailgut cyst, Spinal tumor, Developmental abnormality, Spinal cyst, Case report

## Abstract

Tailgut cysts are rare developmental cysts arising from remnants of the embryological postnatal gut. Despite being frequently located in the presacral space, isolated cases of aberrant locations have been reported, including, perirenal, perianal, and subcutaneous sites, with only two cases of subdural tailgut cysts reported to date. The clinical course is often marked by linear growth, causing compression of the adjacent structures, however malignant transformation with carcinomatous features has been previously described. Hereby the authors describe a case of an intradural extramedullary tailgut cyst in a 33-year-old man presenting with progressive low back pain and signs of autonomic dysfunction, including urinary retention and bowel incontinence. Whole-spine MRI revealed an intrathecal cystic lesion located at L2-L3 level exhibiting hyperintensity on T2-weighted images not enhancing when contrast was administered. Laminectomy followed by tumor excision was performed and pathological analysis confirmed the diagnosis of tailgut cyst.

## 1 Introduction

Tailgut cysts, also known as cystic hamartomas, are rare mucous-secreting congenital lesions that are thought to be derived by a developmental nondisjunction of the embryonic hindgut [1, 2, 3]. These benign lesions tend to be located in the presacral space [4], lying anterior to the sacrum and posterior to the rectum. Occasionally perirenal, perianal, and subcutaneous involvement have been described [5, 6, 7] with intradural tailgut cysts remaining an extremely rare condition with only two cases reported in the literature [8, 9]. Whereas almost exclusively exhibiting a benign behavior, cases of malignant transformation have been described [8, 10, 11]. Given the abundance of epithelioid cells, frequently associated malignant transformation displays carcinomatous features. Hereby an unusual case of a patient with low back pain unresponsive to symptomatic pharmacotherapy and bladder and bowel incontinence is described. Spinal MRI and microscopic analysis confirmed the diagnosis of intradural tailgut cyst without evidence of malignant transformation.

## 2 Case report

**Figure 1:**
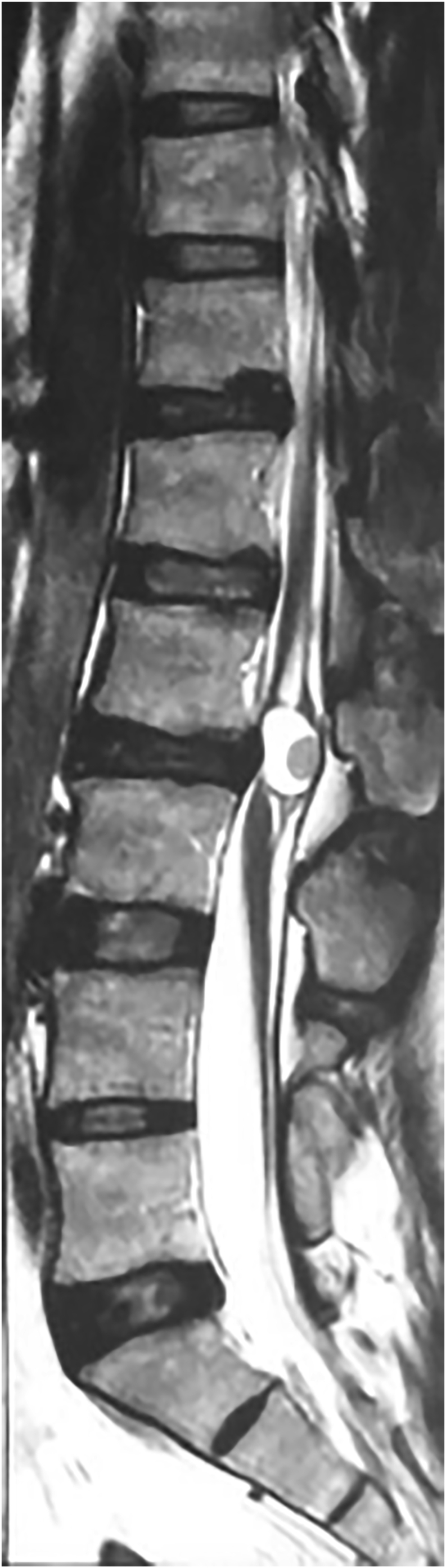
T2-weighted magnetic resonance image showing intradural cystic lesion with an intrinsic nodular component causing compression of the spinal cord.

**Figure 2:**
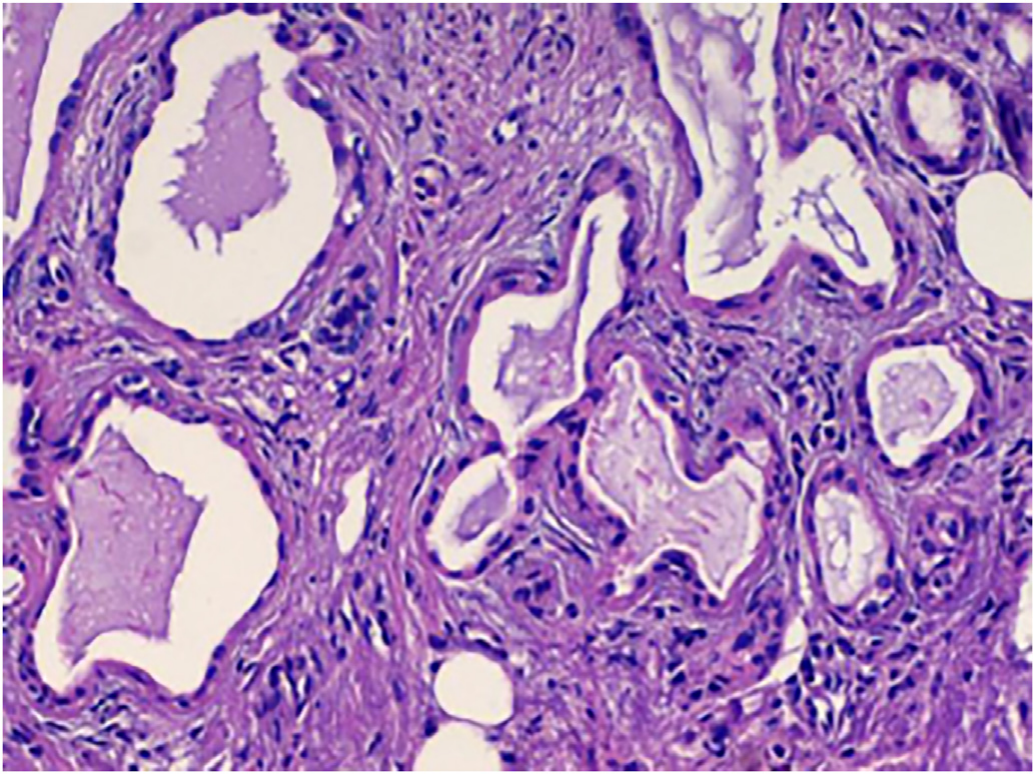
Microphotography revealing mucoid secreting epithelial cells displaying multicystic architecture.

The authors present a case of a 33-year-old man with progressive worsening of low back pain, unresponsive to symptomatic pharmacotherapy without any other relevant past medical history. At admission, the patient referred reduced touch sensation in the lower limbs, more pronounced in the right leg, associated with diffuse paresthesia of the L2-L3 corresponding dermatomal area. Neurological examination showed marked weakness (MRC 3/5 and 4/5 in the left and right leg, respectively), with a slight but present response to deep tendon reflexes stimulation. Anal sphincter insufficiency and urinary retention were also detected. No other neurological deficits were noticed, therefore, a whole-spine magnetic resonance imaging was performed (Figure 1). The scan showed a cystic lesion of the conus medullaris measuring 16 × 10 × 15 mm located at L2-L3 level exhibiting homogeneous hyperintensity on T2-weighted images. A solid nodule located in the posterior region of the cyst did not demonstrate enhancement when gadolinium was administered. A posterior laminectomy of the L2-L4 segment followed by tumor excision was performed. At the opening, the cystic portion of the lesion contained a brownish fluid and the posterior nodule appeared strictly adherent to the thin dural sac. Pathological analysis revealed cystic lining of epithelial-like cells showing mucin secretion capability, confirming the diagnosis of tailgut cyst (Figure 2). The post-operative course was uneventful, and the patient did not experience any surgical complications. He was then transferred to a neurorehabilitation institution and his neurological condition progressively improved. At 6-month follow-up examination, no evidence of lower limbs hyposthenia was noticed and DTR stimulation showed a brisk response. Control spinal MRI did not demonstrate signs of recurrence.

## 3 Discussion

The tailgut or postanal gut is the distalmost part of the embryonic gut, caudal to future anus. It normally reaches its largest diameter on the 35^th^ day of gestation and involutes by the eighth week of embryonic development [2, 3]. Given the proximity of the tailgut to the neural structures, an intra-axial cyst arising from developmental nondisjunction is possible, however, only two cases of intradural formation of tailgut cysts have been reported to date (Table 1) [8, 9]. Developmental cysts are almost invariably located in the presacral space, lying anterior to the sacrum and posterior to the rectum but have also been described in perirenal, perianal, and subcutaneous locations [4, 6, 7]. Due to the rarity of this malformation, real epidemiological data are difficult to obtain since only case reports and small case series are described in the literature [12]. In general tailgut cysts become clinically evident by the fifth decade, showing a marked female preponderance with a 3:1 ratio [13, 14]. However, this condition has also been described in infants and in the fetus, strengthening the theory of a congenital origin [15]. Hjermstad et al. [4] reported the largest series of tailgut cysts to date. They described 53 cases of tailgut cysts localized to the presacral space. Common clinical manifestations included discomfort and pain when sitting, change of stool caliber, fistula formation, infection, and bleeding. However, in approximately half of the cases, the diagnosis was incidental since these patients presented with no evident symptomatology. Although significantly rare, a review of the papers describing intradural tailgut cysts demonstrates peculiar clinical features that these may exhibit [8, 9]. For instance, common manifestations include autonomic dysfunction, especially of the sacral plexus that supplies the hindgut and the pelvic area, weakness and paresthesia in the lower extremities causing gait disturbances.

**Table 1:** Clinical characteristics of patients with intradural extramedullary tailgut cysts.

	Niazi TN et al. [8]	Kemp J [9].	Present case
Age (years)	28	14	33
Sex	F	F	M
Level of the tumor	T10-S1	S1–S4	L2-L3
Follow-up (months)	12	6	6
Clinical presentation	Worsening back pain and gait difficulties, with repeated urinary infections and bowel dysfunction	2-year history of progressive bowel and bladder dysfunction, and increasing lower extremity weakness with abnormal gait	Progressive low back pain with reduced touch sensation in the lower extremities and diffuse dermatomal paresthesia
Neurological examination	Spastic gate without evidence of pathological reflexes, diminished rectal tone, and incomplete bladder emptying	Decreased sensation in the feet to light touch, inability to dorsiflex the right foot and weakness in left dorsiflexion, with positive Babinski reflexes bilaterally	Marked hyposthenia, hyporeflexia in the lower extremities, anal sphincter insufficiency and urinary retention
Radiological findings	T1 isointensity and T2 hyperintensity, with a large nodular enhancing component	Non-enhancing T1 hypointense and T2 hyperintense	T2 hyperintense cystic component with a non-enhancing nodular component
Associated neurological conditions		Anterior sacral meningoceles, tethered sacral cord, and holocord syringomyelia	
Surgical treatment	T10-S4 laminectomy with duraplasty followed by gross total tumor excision	S1–S4 laminectomy, coagulation and transection of the filum terminalis, excision of the lesion	L2-L4 laminectomy with duraplasty followed by gross total tumor excision
Pathological findings	Epithelial cell-lined tissues associated with smooth muscle and adipose tissue, Evidence of strong positivity for synaptophysin and focal positivity for chromogranin, suggesting the carcinoid features	Mucin filled cysts and multiple epithelial types including squamous, transitional, cuboidal, and enteric, with interposed disorganized bundles of smooth muscle	Cystic architecture of epithelial-like cells showing mucin secretion capability
Outcome	Normal dorsiflexion and plantar flexion bilaterally, and improved bladder function	Weakness of foot intrinsics bilaterally, normal sensation and deambulation, and persistent areflexia demonstrated by urodynamic studies	Absence of lower extremities hypostenia and brisk responses to deep tendon reflexes

Grossly, tailgut cysts are soft, multiloculated, and usually well-circumscribed, exhibiting surrounding adherent fibroadipose tissue. Microscopically, these lesions are usually multicystic and lined by a variety of epithelial types, including transitional, squamous, ciliated columnar, and mucinsecreting epithelium; the latter was also apparent in the present case. Disorganized bundles of smooth muscle cells could be seen interposed within the multilocular cysts along with dense mucoid fluid [12]. Although tailgut cysts include all three germ layers several authors consider this developmental abnormality a separate entity from teratoma due to the absence of neural features or bone encroachment [4, 8]. Whereas the majority of tailgut cysts are primarily benign in both behavior and growth, there have been reports of malignant transformation [8, 10, 11]. Because of the abundance of epithelial cells that organize in cystic formations, it is not surprising that carcinomatous features are frequently observed in malignant tailgut cysts. Niazi et al. [8] described a case of intradural tailgut cysts with carcinoid features, in a patient who did not harbor the typical symptoms of carcinoid syndrome, secondary to excessive increase of serotonin secretion causing wheezing, diarrhea, paroxysmal flushing, and heath failure [10, 16].

The differential diagnosis includes a wide variety of conditions occurring in the presacral space, including cystic sacrococcygeal teratoma, duct and gland cyst, pyogenic abscess, neurogenic cyst, and necrotic sacral chordoma. When intradural, spinal cord compression is also noticed, and the additional differential diagnosis must include intervertebral disk herniation with radicular compression, lumbar stenosis, and intra-axial tumors. Magnetic resonance imaging with contrast administration represents the gold standard to define the relation of the tumor with adjacent structures and to initially differentiate benign from malignant lesions [17]. Generally, these lesions range from 2 to 14 cm in maximum diameter, depending on their location: intradural tailgut cysts usually become clinically evident prior to presacral cysts, therefore prompting intervention when maximum diameter reaches 2–2.5 cm [9]. Due to the abundant mucoid component, these lesions exhibit homogeneous hypodensity on T1 weighted images and high signal intensity on T2-weighted images [18]. Nevertheless, malignancy should be suspected if there is evidence of a heterogeneous thickening with irregular margins or avid enhancement is seen of the soft tissues [8, 19].

## 4 Conclusion

In the present report, a rare case of intradural extramedullary tailgut cyst is described. As a result of its intra-axial location, the lesion caused atypical clinical manifestations secondary to spinal cord and nerve root compression. The patient presented with marked hyposthenia and diffuse paresthesia and was unresponsive to symptomatic pharmacotherapy for low back pain. Furthermore, autonomic dysfunction was noticed during examination with signs of urinary retention and anal sphincter insufficiency, therefore prompting urgent decompressive surgery followed by tumor excision. The pathological analysis confirmed the diagnosis of tailgut cyst with mucin secreting epithelial cells.

The patient's written informed consent was collected prior to data collection and available on reasonable request.

## Declarations

### Author contribution statement

All authors listed have significantly contributed to the investigation, development and writing of this article.

### Funding statement

This research did not receive any specific grant from funding agencies in the public, commercial, or not-for-profit sectors.

### Data availability statement

Data will be made available on request.

### Declaration of interests statement

The authors declare no conflict of interest.

### Additional information

No additional information is available for this paper.
